# The Effect of Vitamin D_3_ Supplementation on Physical Capacity among Active College-Aged Males

**DOI:** 10.3390/nu12071936

**Published:** 2020-06-30

**Authors:** Sylwester Kujach, Dariusz Lyzwinski, Maciej Chroboczek, Dawid Bialowas, Jedrzej Antosiewicz, Radoslaw Laskowski

**Affiliations:** 1 Faculty of Physical Education, Department of Physiology, Gdansk University of Physical Education and Sport, Gorskiego 1, 80-336 Gdansk, Poland; maciej.chroboczek@gmail.com (M.C.); bialowasdawid@gmail.com (D.B.); radoslaw.laskowski@awf.gda.pl (R.L.); 2 Department of Sport and Physical Education, Medical University of Gdansk, M. Skłodowskiej-Curie 3a, 80-210 Gdansk, Poland; dariusz.lyzwinski@gumed.edu.pl; 3 Faculty of Health Sciences, Department of Bioenergetics and Physiology of Exercise, Medical University of Gdansk, Debinki 1, 80-211 Gdansk, Poland; jedrzej.antosiewicz@gumed.edu.pl

**Keywords:** vitamin D, physical capacity, vitamin D_3_ supplementation, anaerobic capacity, aerobic capacity

## Abstract

Vitamin D_3_ supplementation can affect strength and power; however, the effect on both aerobic and anaerobic performance remains unclear. Here, we investigate the effects of eight weeks of a high dose of vitamin D_3_ supplementation and its impact on circulating 25-hydroxyvitamin D (25-OH-D_3_) concentrations and selected indicators of physical capacity. Subjects (*n* = 28, age 21.1 ± 1.6) were divided into two groups: supplemented (SUP), which was given 6000 IU of vitamin D_3_ daily for eight weeks; and placebo group (PLA). Serum 25-OH-D_3_ concentrations were determined in pre- and post-intervention. Aerobic ($\dot{V}$O_2max_ test) and anaerobic (Wingate Anaerobic Test) capacity were determined before and after the supplementation. The mean baseline concentration of 25-OH-D_3_ was recognized as deficient (20 ng/mL) and significantly increased over time in the supplemented group (*p* < 0.01, η^2^ = 0.86), whilst it remained unchanged in the placebo group. Moreover, the supplementation caused a significant improvement in maximal aerobic (*p* < 0.05, η^2^ = 0.27) and anaerobic power (*p* < 0.01, η^2^ = 0.51) whereas no changes were observed in PLA group. The $\dot{V}$O_2max_ differences were also significant in the supplemented group (*p* < 0.05). In summary, the changes in aerobic and anaerobic capacity observed in this study were associated with a serum concentration of 25-OH-D_3_. Our data imply that vitamin D_3_ supplementation with a dose of 6000 IU daily for eight weeks is sufficient to improve physical capacity and vitamin D_3_ status.

## 1. Introduction

Sport and recreational activity-induced adaptation includes changes in skeletal muscle, such as biogenesis of mitochondria, induction of antioxidant enzymes, etc. [1] Exercise also induces changes in other tissues such as the heart, adipose, bones, arteries, and lungs [2]. Interestingly, there is an increasing number of studies demonstrating that exercise-induced adaptation is related to vitamin D status [3,4]. Conversely, several studies indicate that many athletes and non-athletes are vitamin D deficient [4,5]. Vitamin D deficiency is defined as a concentration of 25-OH-D_3_ lower than 20 ng/mL while optimal concentration is 30–60 ng/mL [6]. Low vitamin D status is a result of sunlight limitations due to geographical location, lifestyle associated with spending most of the time indoors (both during work and in free time) and the so-called Western diet, i.e., a diet poor in natural exogenous sources of vitamin D [6]. Thus, international health organizations recommend 400 to 6000 IU/day vitamin D supplementation, depending on age and health status, so the concentration of 25-OH-D_3_ reaches the optimal level [6]. It has been reported that athletes and non-trained controls with vitamin D deficiency had smaller hearts compared with vitamin D sufficient subjects [7]. As cardiac output is strictly related to submaximal and maximal oxygen consumption, one can expect that correcting vitamin D deficiency could have a positive impact on $\dot{V}$O_2max._ Consistently reports demonstrate a positive association between serum 25-OH-D_3_ and $\dot{V}$O_2max_ [8]. Conversely, no such association was observed in another study [9]. What is more, oxygen uptake is related to pulmonary function [2]. There are limited data as to whether vitamin D status can modify lung function in human subjects especially during exhaustive exercise; however, vitamin D deficiency has been shown to cause deficits in pulmonary function associated with lower lung volume compared to in vitamin D sufficient animals [10,11]. Certainly, the effects of vitamin D on lung function can be related to respiratory muscles [10,12], whereas decreased efficiency of respiratory muscle contraction can significantly limit physical capacity and exercise performance [13].

Vitamin D receptors (VDR) have been found in both nuclei and membranes of the human skeletal muscle cells [14]. Through its receptor, vitamin D_3_ modifies many processes including inhibition of skeletal muscle atrophy, anti-inflammatory, anti-apoptotic, cell differentiation, and proliferation functions [14,15]. It can also modify skeletal muscle metabolism and antioxidant potential [14,16,17,18,19]. It has been shown that vitamin D status could modulate muscle strength and power measured by maximum number of repetitions (bench press, leg extension, and flexion exercises, or 10–20 s sprint ability) among the elderly, active individuals, and athletes [3,8,20,21].

Conversely, vitamin D deficiency has been associated with impaired muscle action, including muscle weakness [22], sarcopenia development [23], and decreased muscle strength [24]. There is an increasing amount of evidence indicating a positive effect of vitamin D supplementation on physical performance; however, the results from studies analyzing the effect of vitamin D supplementation on aerobic capacity-reflected by $\dot{V}$O_2max_ level are still ambiguous [9,25,26,27].

Exogenous vitamin D supplementation is considered to be an effective approach to improve vitamin D status [28].

Given this evidence, we hypothesized that aerobic and anaerobic physical capacity would improve in response to vitamin D supplementation and be related to improvements in the respiratory function. Here, we investigated whether eight weeks of high dose vitamin D_3_ supplementation affects circulating 25-OH-D_3_ concentrations and evaluate if the improved status of vitamin D can be related to changes in physical performance. 

## 2. Materials and Methods 

A total of 28 healthy, male subjects participated in the study. Subjects were divided into two groups: supplemented (SUP; *n* = 14, mean age 21.7 ± 1.8 years) and placebo group (PLA; *n* = 14, mean age 20.5 ± 1.4 years), based on their physical fitness level ($\dot{V}$O_2max_) and anaerobic capacity. Initial physical capacity level as well as vitamin D concentration did not differ between supplemented and placebo groups. All the subjects provided written informed consent before the study procedures. All the procedures were approved by the Bioethical Committee of the Regional Medical Society (KB-3/14). The study was conducted in accordance with the Declaration of Helsinki. 

### 2.1. Study Design

**Figure 1:**
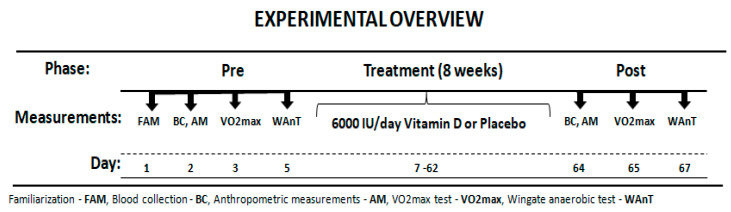
Experimental Overview.

The participants reported to the laboratory on three separate occasions before and after treatment. Before starting the experiment, the subjects were interviewed about eating habits and level of physical activity. Additionally, participants were asked to come to the laboratory for a familiarization, to learn about the testing procedures. Next, they completed blood collection, anthropometric measurements and aerobic ($\dot{V}$O_2max_) and anaerobic assessment (Wingate test). After one day the main intervention session followed. All participants performed the same battery of exercise tests before and after the treatment period. Researchers carrying out exercise tests were not informed about the type of treatment. An overview of the experimental protocol is presented in Figure 1. All the physical capacity tests took place in the morning 2 h after light breakfast.

### 2.2. Anthropometric Measurements 

Body mass (BM) and body composition were analyzed using a multi-frequency impedance plethysmograph body composition analyzer (In Body 720, Biospace, Seoul, Korea). The participants had voided their bladders and bowels prior to the analysis. The measurements were performed in the standing position that is recommended in the manuals and the subjects wore only briefs. The analyzer accurately measures body water and body composition, including fat mass, free fat mass, skeletal muscle mass, and soft lean mass [29].

### 2.3. Supplementation

Subjects from the supplemented group were given 6000 IU of vitamin D_3_ daily in capsule form (2000 IU per capsule), whereas the placebo group received identical-looking capsules containing sunflower oil. Capsules were administered in a single-blind design. All participants were asked to take three capsules per day in the morning for eight weeks. The supplementation procedure was carried out from January to the beginning of March. The participants were medical university students (not professional athletes) and their physical activity level and diet were determined during the first interview. One month before and during the experiment, participants did not take any vitamin or other supplements. The participants took part in 3 h per week of supervised physical activity and diet did not differ from the accepted nutrition standards. Diets were not strictly standardized but the participants were instructed by the dietitian to maintain their nutritional habits throughout the whole experiment.

### 2.4. Maximal Oxygen Uptake-$\dot{V}$O_2max_ Test

The participants performed a graded ergometry test on a cycle ergometer (884E Sprint Bike, Monark, Vansbro, Sweden). The ergometer seat height was individually adjusted and the participants were allowed a 5 min warm-up period at an intensity of 1.5 W·kg^−1^ with a pedaling cadence of 60 rpm. After the warm-up period, work rate was increased by 25 W·min^−1^ until volitional exhaustion. Breath by breath pulmonary gas exchange was measured using MetaMax 3B (Cortex Biophysik GmbH, Leipzig, Germany). During $\dot{V}$O_2max_ test, maximal lung minute ventilation (VE_max_ mL·min^−1^), maximal breath frequency (BF_max_ 1·min^−1^), and $\dot{V}$O_2max_ were determined when at least two of the following criteria were met: (1) achievement of 90% of age-predicted peak heart rate (220-age), (2) a rating of perceived exertion scale (RPE) of 19 or 20, and (3) respiratory exchange ratio (RER) exceeded 1.05 [30].

### 2.5. Anaerobic Capacity Measurement—Wingate Anaerobic Test

After a standard warm-up, all subjects performed a 30 s “all-out” supramaximal test on a mechanically braked cycle ergometer (884E Sprint Bike, Monark, Vansbro, Sweden). The test was initiated from a dead stop with the resistance equal to 75 g·kg body mass^−1^ (corresponding to 7.5% of an individual’s body mass) preset on the ergometer’s friction belt [31,32]. The obtained results were analyzed for maximal anaerobic power (MAnP) and work output.

### 2.6. Vitamin D Determination

The assay was performed on the IDS-iSYS Multi-Discipline Automated Analyzer. 10 µL of serum aliquots were automatically pipetted and subjected to a pre-treatment step with NaOH (part of the reagent used for CLIA and ELISA methods) to denature the DBP inside the IDS-iSYS Multi-Discipline Automated Analyzer. This assay is aligned to the NIST SRM 2972. The measurement range of this assay is 7–125 ng/mL (information of the manufacturer). The IDS-iSYS 25(OH)DS Control Set (IS-2730S) was used for quality control [33].

### 2.7. Statistical Analyses

The statistical analyses were performed using STATISTICA 13.0 software (Statsoft, Tulsa, OK, USA). All data are expressed as mean and standard deviation (SD), or standard error of mean (SEM). The normality of data distribution was established using the Shapiro-Wilk W-test. The level of significance was set as *p* < 0.05 for all of the analyses. Additionally, a two-way analysis of variance (ANOVA) with repeated measures was used to investigate the significance of differences between groups and time. Significant main effects were further analyzed using the Bonferroni or Tukey post hoc test. The effect size (η^2^) has also been calculated. The value of η^2^ has been interpreted as follows: 0.1 a small effect, 0.3 a medium effect, and 0.5 a large effect, as previously described [32]. Changes (delta) in both groups were compared using an independent samples *t*-test or a U-Mann–Whitney test, according to the data distribution. Correlations between variables were evaluated using the Pearson correlation coefficient.

## 3. Results

### 3.1. Subject Characteristics 

All subjects completed the study. The anthropometric and physical activity parameters are presented in Table 1. At baseline, there were no significant differences in basic anthropometric characteristics or in aerobic performance between the groups.

### 3.2. Vitamin D Status

**Figure 2:**
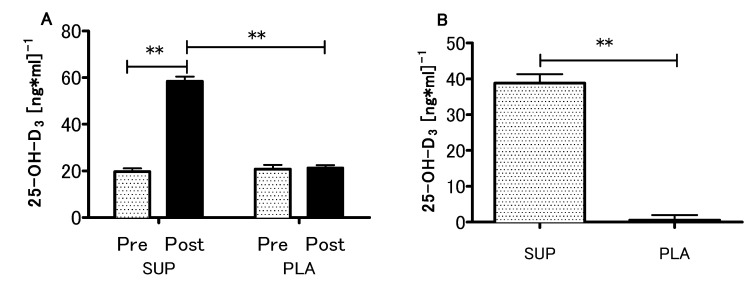
Effect of Vitamin D supplementation on serum 25-OH-D_3_ concentration (**A**), and contrast between supplemented (SUP) versus placebo (PLA) deltas (post-pre) (**B**). Values are means. Error bars indicate ± SEM. ** = *p* < 0.01.

At the baseline, the mean serum concentration of 25-OH-D_3_ in both supplemented and control groups was 20.2 ± 6.2 ng/mL (~50 nmol/L), which was recognized as a deficiency [34]. What is more, initial 25-OH-D_3_ levels did not differ between supplemented and placebo groups (19.6 ± 5.4 vs 20.7 ± 6.8 ng/mL). In the supplemented group, the serum 25-OH-D_3_ concentration increased significantly after the intervention (initial 19.6 ± 5.4 ng/mL, after intervention 58.4 ± 7.3 ng/mL, ~200% increase, *p* < 0.001), while in the placebo group only non-significant changes were observed (initial 20.7 ± 6.8 ng/ml, after intervention 21.2 ± 4.7 ng/mL, ~2.5% increase, *p* > 0.05) (Figure 2A). There was a significant interaction between the groups (SUP/PLA) and time (PRE/POST) factors when we performed a two-way ANOVA with repeated measures (*p* < 0.01, η^2^ = 0.86; F (1.26) = 172.1). The delta 25-OH-D_3_ difference was significantly more positive in the SUP than in the PLA group (z = 4.47; *p* < 0.001) (Figure 2B).

### 3.3. Effect of Vitamin D Supplementation on Aerobic Capacity 

**Figure 3:**
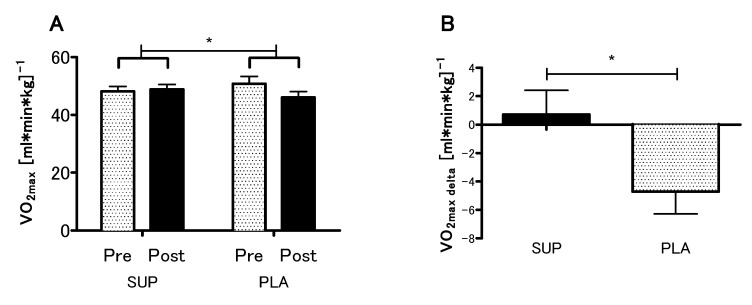
Effect of vitamin D supplementation on $\dot{V}$O_2max_ (**A**), and contrast between SUP versus PLA deltas (post-pre) (**B**). Values are means. Error bars indicate ± SEM. * = *p* < 0.05.

**Figure 4:**
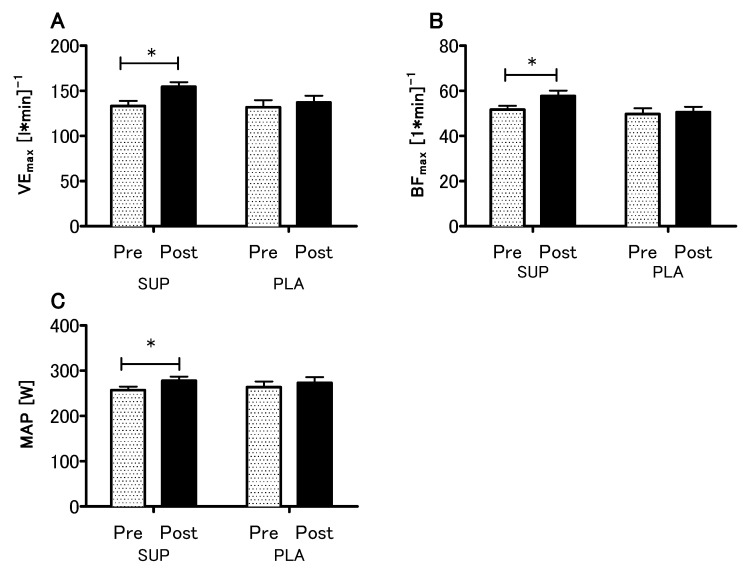
Effect of vitamin D supplementation on maximal minute ventilation VE_max_ (**A**) maximal breath frequency BF_max_ (**B**) and maximal aerobic power MAP (**C**) recorded during the $\dot{V}$O_2max_ test. Values are means. Error bars indicate ± SEM (standard error of mean). * = *p* < 0.05.

The analyses revealed no statistical differences between the SUP and PLA groups in $\dot{V}$O_2max_ (*p* = 0.79), VE_max_ (*p* = 0.99), BF_max_ (*p* = 0.92) and MAP (*p* = 0.96) for the pre-sessions. There was significant interaction between group and time (SUP/PLA) × (PRE/POST) factors when we performed a two-way ANOVA with repeated measures for $\dot{V}$O_2max_ (*p* < 0.05, η^2^ = 0.17; F (1.26) = 5.54) (Figure 3A). Next, we performed contrast analyses between SUP (post-pre) versus PLA (post-pre). The delta $\dot{V}$O_2max_ difference was significantly different between groups t (26) = 2.35; *p* < 0.05, unpaired *t*-test (Figure 3B). Moreover, there was a significant main effect of time in the VE_max_, BF_max_ and MAP (*p* < 0.01, η^2^ = 0.26; F (1.26) = 9.33., *p* < 0.05, η^2^ = 0.12; F (1,26) = 3.88 and *p* < 0.01, η^2^ = 0.27; F (1.26) = 9.64, respectively), whereas neither main effect of group nor interaction of the factors was significant. The analyses revealed a significant increase in the VE_max_, BF_max_, and MAP among the SUP group (Figure 4).

### 3.4. Effect of Vitamin D Supplementation on Anaerobic Capacity

**Figure 5:**
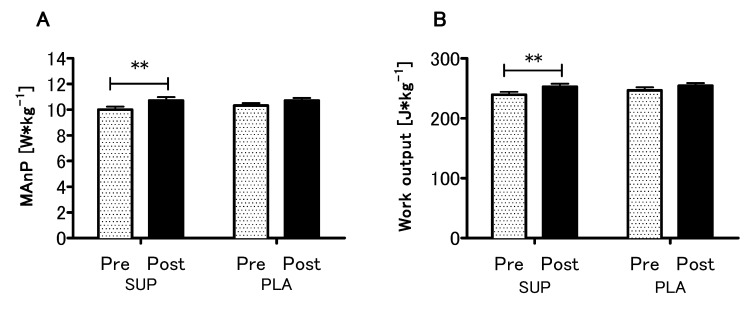
Effect of vitamin D supplementation on maximal anaerobic power MAnP (**A**) and work output (**B**). Values are means. Error bars indicate ± SEM (standard error of mean). ** = *p* < 0.01.

Analyzing the effect of Vitamin D supplementation on anaerobic capacity parameters, a significant main effect of time in the MAnP and Work (*p* < 0.01, η^2^ = 0.51; F (1,26) = 28.10 and *p* < 0.01, η^2^ = 0.52; F (1,26) = 28.54, respectively) were found, whereas neither main effect of group nor interaction of the factors was significant (Figure 5A,B). Detailed results of the physical capacity tests in the present study are listed in Supplementary Table S1.

### 3.5. Correlation Analyses 

**Figure 6:**
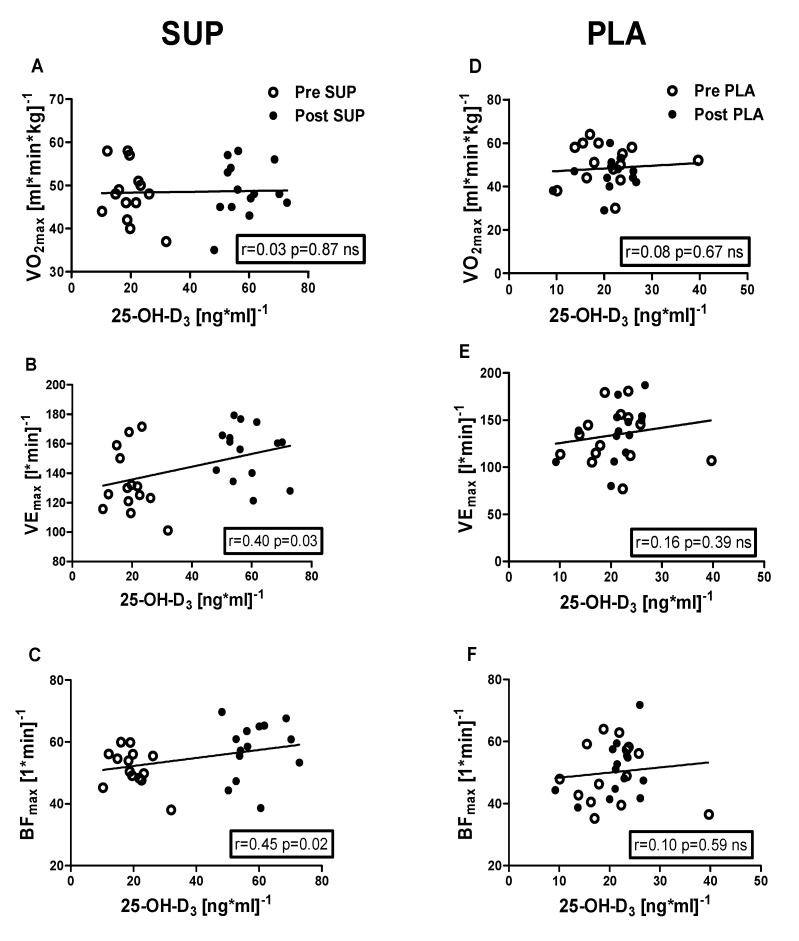
Association between 25-OH-D_3_ and $\dot{V}$O_2max_, VE_max_, and BF_max_, of pre- and post-intervention in the SUP and PLA groups. Significant positive correlations between 25-OH-D_3_ and VE_max_ (**B**), 25-OH-D_3_, and BF_max_ (**C**) were found in the SUP group. Correlations between 25-OH-D_3_ and $\dot{V}$O_2max_ (**D**), VE_max_ (**E**), and BF_max_ (**F**) in PLA group were not found. Data are presented as open circles: pre, and closed circles: post SUP or PLA. ns, non-significant.

**Figure 7:**
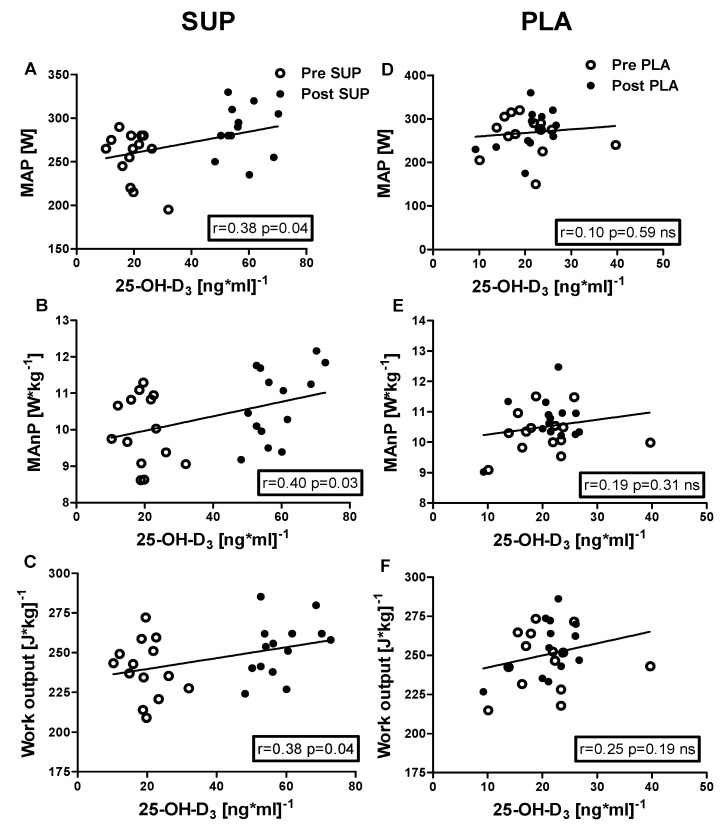
Association between 25-OH-D_3_ and MAP, MAnP and Work output, of pre- and post-intervention in the SUP and PLA groups. Significant positive correlations between 25-OH-D_3_ and MAP (**A**), 25-OH-D_3_ and MAnP (**B**), and between 25-OH-D_3_ and Work output (**C**) were found in the SUP group. Correlations between 25-OH-D_3_ and MAP (**D**), MAnP (**E**), and Work output (**F**) in PLA group were not found. Data are presented as open circles: pre, and closed circles: post SUP or PLA. ns, non-significant.

Positive correlations were identified between 25-OH-D_3_ concentration and the following: VE_max_ (r = 0.40, *p* = 0.03); BF_max_ (r = 0.45, *p* = 0.02); MAP (r = 0.38, *p* = 0.04); MAnP (r = 0.40, *p* = 0.03), and Work output (r = 0.38, *p* = 0.04) in the pre- and post-intervention measurements in the SUP group. Whereas neither association between 25-OH-D_3_ and physical capacity parameters was significant in PLA group (Figure 6 and Figure 7).

## 4. Discussion

In the present study, we demonstrated that supplementation improves vitamin D levels and has a positive impact on aerobic and anaerobic performance. Our data imply that the participants’ low vitamin D status negatively influences their performance despite their young age and the fact that their aerobic physical fitness level was fair. The present data also revealed that aerobic physical capacity can be modulated by 25-OH-D_3_ concentration. We found an increase in post-intervention pulmonary parameters (VE_max_ and BF_max_) as well as aerobic power. Although we did not observe post supplementation $\dot{V}$O_2max_ increase, when deltas of $\dot{V}$O_2max_ are compared they became significantly different. These data are in agreement with previous studies demonstrating lower aerobic performance which was corrected by UV radiation or vitamin D supplementation [35]. Besides, the winter season diminishes physical activity, which may result in a decrease in aerobic fitness, similar to immobilization [35,36,37] and physical capacity level was shown to be highly seasonal [35]. The results from the maximal bicycle exercise test among Norwegian men showed a peak in August, nadir in winter and decline starting in the autumn [38]. Further, a study of Koch and Raschka (2000) demonstrated the seasonality of physical performance, indicating maximal oxygen uptake peak in the late summer [39]. Also, data from professional athletes displayed maximal oxygen uptake during the summer months [35,40]. The reduced level of $\dot{V}$O_2max_ in the placebo group after the intervention (from January to the beginning of March) seems to confirm these assumptions. Additionally, our results indicate that supplementation can compensate for $\dot{V}$O_2max_ decrease in the season of reduced physical activity, possibly by an improvement of skeletal muscle and respiratory function. However, we did not control physical activity via accelerometers or the GPS devices of our participants during the experiment, which should be considered in future studies. Experiments on cell culture demonstrated that skeletal muscle cells treated with 1α,25(OH)_2_D_3_, have shown increased oxygen consumption and increase ATP production [14,18]. Moreover, activity of citrate synthase (40% increase) as well as protein content of PGC-1α, a transcriptional coactivator, was higher in the paraspinal muscle after vitamin D_3_ supplementation [14]. This data implies that vitamin D supplementation improves the function of mitochondria in our participants. However, we did not observe any association between $\dot{V}$O_2max_ and serum 25-OH-D_3_ concentration. Accordingly, no associations between 25-OH-D_3_ concentration and $\dot{V}$O_2max_ were stated by Książek et al. (2016) in professional athletes and resistance-trained participants [26,41]. There was also no significant association between 25-OH-D_3_ with $\dot{V}$O_2max_ in 52 professional ice hockey players [9]. In contrast to our study, Adrestani et al. (2013) observed a significant positive correlation between 25-OH-D_3_ concentration and $\dot{V}$O_2max_ in both men and women over a broad range of age (20–73 years) and serum 25-OH-D_3_ levels (10–82 ng/mL). Interestingly, the effect was greatest among those with low levels of physical activity [25]. Also, Mowry et al. (2009) examined the relation of cardiorespiratory fitness $\dot{V}$O_2max_ and 25-OH-D_3_ in young healthy women, showing a positive association between both factors [27]. Although we did not find similar results in the literature analyzing the respiratory parameters during graded exercise tests following supplementation with vitamin D, it seems that the changes in VE_max_ and BF_max_ are associated with improved lung function and/or structure as well as an increase in respiratory muscle function (strength and/or efficiency) [11]. An increase in VE_max_ and BF_max_ are considered as an adaptive response to training and well-trained persons are characterized by higher values of these two parameters compared to untrained. Therefore, the increase in VE_max_ and BF_max_ via lung and respiratory muscle function are possibly one of the beneficial effects of vitamin D supplementation.

The mechanisms by which vitamin D modulates aerobic capacity could also be related to cardiac function and structure as it has been observed that vitamin D deficiency is associated with low left ventricle mass and other heart measures [7]. In addition, vitamin D can modulate arterial oxygen content, regulation of muscle blood flow, and oxygen uptake and utilization by exercising muscles, whereas anaerobic capacity may be shifted by the regulation of calcium metabolism, muscle protein synthesis, neuromuscular efficiency and/or insulin signaling [35,42,43,44].

Vitamin D elevated through supplementation is potentially a factor that leads to improved muscle strength and power [45]. It has been demonstrated that vitamin D will interact with calcium in muscle function, plasticity, and insulin signaling [17]. Additionally, the change in vitamin D concentration also modulates its receptors at the expression and activation levels [46,47], thus affecting muscle mass [15,46], the relative number and the cross-sectional area of type II muscle fibers, and neuromuscular coordination [48]. The results of Koundourakis et al. (2014) indicated that serum 25-OH-D_3_ concentration is positively correlated with anaerobic performance such as 10 and 20 s sprints as well as jumps test [49]. Moreover, Close et al. (2013) have shown a significant elevation in vertical jump, 10 m sprint results, and a trend for improved bench press and back squat [50]. The authors suggest that vitamin D may play a regulatory role in the insulin-like growth factor-1 (IGF-1) pathway via a transcription enhancing role for insulin-like growth factor binding protein-3 IGFBP-3 [51]. We also observed a significant increase in maximal anaerobic power and work output after vitamin D treatment. Furthermore, a significant positive correlation between 25-OH-D_3_ concentration and both anaerobic parameters in the supplemented group was observed. Thus, we cannot rule out that the improvement in anaerobic capacity resulted from the vitamin D supplementation along with induction in anabolic pathways in skeletal muscle [14]. 

It is important to note that our participants are characterized by a low baseline 25-OH-D_3_ concentration. Due to the angle of sunlight, the synthesis of vitamin D is limited in the winter months in latitudes above 35°, causing a clear seasonality of 25-OH-D_3_ concentrations [35]. The geographical location of Poland (54° N), and associated with it the ultraviolet (UV) radiation, ensure sufficient vitamin D skin synthesis only from April to September [52,53]. Considering that skin synthesis is a source of 80–90% of vitamin D in the body [54,55], there is a risk of its insufficient production in the remaining months of the year [52,53]. This confirms the hypothesis concerning the impact of latitude (54.2° N) and season (winter) on 25-OH-D_3_ concentration and insufficiency and/or deficiency [56,57]. Moreover, we found that eight weeks of vitamin D_3_ supplementation (6000 IU·day^−1^) significantly increases (~200%) 25-OH-D_3_ serum concentration in young healthy males. It is suggested that skeletal muscle may require higher 25-OH-D_3_ concentrations, to achieve beneficial changes in physical performance [35]. Through the administration of a higher dose of vitamin D, the concentration of 25-OH-D_3_ reaches above 40 ng/mL-(autocrine or paracrine effect) in the circulation [34,35,50]. Therefore, we can postulate that the eight weeks of 6000 IU·day^−1^ vitamin D_3_ supplementation are sufficient to eliminate the deficiency among young participants. Our data are consistent with other studies showing significant systemic 25-OH-D_3_ concentration increase (~100%) following 6–8 weeks vitamin D_3_ supplementation (5000–6000 IU/day) in a similar location/sun exposure among physically active participants [34,54,58]. Recent reports have shown that vitamin D deficiency is widespread across Europe and at prevalence rates that meet the pandemic criteria [59]. Thus, it is possible that if the experiment was performed on subjects with better vitamin D status the beneficial effects of supplementation would not be observed. Although the results of statistical analysis showed a significant effect in time only (MAP., VEmax., BFmax., MAnP., Work output), we did not record any significant changes in physical performance in the placebo group. Therefore, significant changes in the supplemented group and their absence in the placebo group may suggest a mild beneficial effect of Vitamin D in maintaining or even increasing selected indicators of physical fitness in young people. 

## 5. Conclusions

In summary, the current findings indicate that the improvement of aerobic and anaerobic capacity may be associated with vitamin D_3_ status. Moreover, eight weeks of vitamin D_3_ supplementation with a dose of 6000 IU per day significantly increases 25-OH-D_3_ serum concentration and was an efficient treatment of vitamin D_3_ deficiency among young healthy males.

## Figures and Tables

**Table 1:** Demographic and clinical characteristics.

Variable	SUP (*n* = 14)		PLA (*n* = 14)	
	Pre	Post	Pre	Post
Age (y)	21.7 ± 1.8	22.0 ± 1.8	20.5 ± 1.4	20.7 ± 1.1
Height (cm)	180.3 ± 7.4	180.3 ± 7.4	183.2 ± 7.1	183.3 ± 7.4
Weight (kg)	76.7 ± 9.0	77.1 ± 8.6	75.9 ± 7.0	75.5 ± 7.1
Fat (%)	14.4 ± 4.4	14.7 ± 4.4	13.0 ± 2.7	12.8 ± 3.2
Fat (kg)	11.3 ± 4.3	11.6 ± 4.2	9.9 ± 2.9	9.8 ± 3.3
FFM (kg)	65.3 ± 5.8	65.5 ± 5.8	65.9 ± 4.8	65.7 ± 4.7
BMI (kg·m^−2^)	23.6 ± 3.1	23.8 ± 2.8	22.6 ± 1.9	22.4 ± 1.9
TBW (kg)	47.8 ± 5.8	47.9 ± 4.2	48.3 ± 3.5	48.1 ± 3.4

SUP: supplemented group; PLA: placebo group; values are mean ± SD expressed in absolute or relative values; Fat (%): fat percentage; Fat (kg): fat mass; FFM: free fat mass; BMI: body mass index; TBW: total body water.

## Supplementary Materials

The following are available online at https://www.mdpi.com/2072-6643/12/7/1936/s1, Table S1: The effect of Vitamin D supplementation on aerobic and anaerobic capacity.
