# Supplementary material for: The Effect of Vitamin D3 Supplementation on Physical Capacity among Active College-Aged Males
Source: Nutrients. 2020 Jun 30;12(7):1936. doi: 10.3390/nu12071936 (PMC7400321; doi:10.3390/nu12071936)
Supplement: Supplementary file 1 [file nutrients-12-01936-s001.pdf]

Supplementary material

Table 1. The effect of Vitamin D supplementation on aerobic and anaerobic capacity.

Variables	PLA		SUP	
	Pre	Post	Pre	Post
25-OH-D_3 [ng*ml^{-1}]	20.7 ± 6.8	21.2 ± 4.7	19.6 ± 5.4	58.4 ± 7.3[ab]
25-OH-D_3 delta[ng*ml^{-1}]	0.5 ± 5.4		38.8 ± 9.4[b]	
VO_{2max} [ml*min*kg]$^{-1}$	50.8 ± 9.5	46.1 ± 7.6[a]	48.1 ± 6.4	48.9 ± 6.3
VO_{2max} delta [ml*min*kg]$^{-1}$	-4.71± 5.8		0.71 ± 6.3[b]	
VE_{max}[l*min]$^{-1}$	131.9 ± 29.6	137.0 ± 28.5	133.3 ± 21.1	154.6 ± 18.4[a]
BF_{max}[1*min]$^{-1}$	49.7 ± 9.9	50.6 ± 8.8	51.7 ± 5.8	57.7 ± 9.1[a]
MAP [W]	263.9 ± 46.2	273.2 ± 45.7	257.1 ± 28.4	277.8 ± 33.5[a]
MAnP [W*kg^{-1}]	10.3 ± 0.7	10.7 ± 0.8	9.9 ± 0.9	10.7 ± 0.9[a]
Work output [J*kg^{-1}]	246.9 ± 18.7	254.3 ± 17.6	239.6 ± 17.4	252.8 ± 17.7[a]

PLA: placebo group; SUP: supplemented group; values are mean ± SD expressed in absolute or relative values; 25-OH-D_3: 25-hydroxyvitamin D; VO_{2max}: maximal oxygen uptake; VEmax: maximal lung minute ventilation; BFmax: maximal breath frequency; MAP: maximal aerobic power; MAnP: maximal anaerobic power.

a significant differences from pre-treatment.

b significant differences between groups.
